# Study of a Microfluidic Chip Integrating Single Cell Trap and 3D Stable Rotation Manipulation

**DOI:** 10.3390/mi7080141

**Published:** 2016-08-12

**Authors:** Liang Huang, Long Tu, Xueyong Zeng, Lu Mi, Xuzhou Li, Wenhui Wang

**Affiliations:** State Key Laboratory of Precision Measurement Technology and Instrument, Department of Precision Instrument, Tsinghua University, Beijing, China; huangl14@mails.tsinghua.edu.cn (L.H.); tulongchina@126.com (L.T.); zengxueyong12@163.com (X.Z.); 503430576@qq.com (L.M.); xz-li13@mails.tsinghua.edu.cn (X.L.)

**Keywords:** 3D cell rotation, single cell manipulation, cell trap, dielectrophoresis (DEP), microfluidics

## Abstract

Single cell manipulation technology has been widely applied in biological fields, such as cell injection/enucleation, cell physiological measurement, and cell imaging. Recently, a biochip platform with a novel configuration of electrodes for cell 3D rotation has been successfully developed by generating rotating electric fields. However, the rotation platform still has two major shortcomings that need to be improved. The primary problem is that there is no on-chip module to facilitate the placement of a single cell into the rotation chamber, which causes very low efficiency in experiment to manually pipette single 10-micron-scale cells into rotation position. Secondly, the cell in the chamber may suffer from unstable rotation, which includes gravity-induced sinking down to the chamber bottom or electric-force-induced on-plane movement. To solve the two problems, in this paper we propose a new microfluidic chip with manipulation capabilities of single cell trap and single cell 3D stable rotation, both on one chip. The new microfluidic chip consists of two parts. The top capture part is based on the least flow resistance principle and is used to capture a single cell and to transport it to the rotation chamber. The bottom rotation part is based on dielectrophoresis (DEP) and is used to 3D rotate the single cell in the rotation chamber with enhanced stability. The two parts are aligned and bonded together to form closed channels for microfluidic handling. Using COMSOL simulation and preliminary experiments, we have verified, in principle, the concept of on-chip single cell traps and 3D stable rotation, and identified key parameters for chip structures, microfluidic handling, and electrode configurations. The work has laid a solid foundation for on-going chip fabrication and experiment validation.

## 1. Introduction

Biological cells need to be manipulated in many scenarios, such as cell characterization, sorting, injection, and enucleation [1,2,3,4,5]. For example, in enucleation, the chromosome of a recipient cell in the animal cloning process needs to be taken out by aspiration, during which the cell has to be rotated around normally by hand, such that the chromosome is oriented upwards for subsequent identification and pipette aspiration. Over the years, efforts have been continuing in the development of different techniques to rotate cells [6,7,8]. At present, cell rotation methods mainly include mechanical means, optical tweezers, magnetic means, and electrical means. Mechanical means involve direct contact between the manipulator probes and cell surface, which poses the risk of damaging the cell structure, as the cell is quite delicate and sticky [9,10]. Additionally, the control precision that can be reached is lower than that of other methods. Optical tweezers can capture and move single cells easily using high-power laser, but are unable to rotate cells in 3D unless high-cost and sophisticated optical instruments are deployed [11,12,13]. Magnetic means could manipulate cells on the premise that cells should be embedded with magnetic nano-particles before moving or rotating under a magnetic field. The complexity of cell sample pretreatment has restricted the efficiency of experiments, and embedded magnetic nano-particles could affect the inherent cellular biological activities [14,15,16,17]. In contrast, electrical methods were widely accepted by researchers because of convenience, low cost, and controllability. In addition, dielectrophoresis (DEP) is one of the most conspicuous methods, which can achieve a high precision and efficiency of operating a single or group of cells [18,19,20,21,22,23].

Recent advances in MEMS technology have enabled miniaturized devices to be fabricated at low cost and high efficacy. DEP methods, combined with microfluidic technology, are applied more rapidly than before in biological fields, such as for cell sorting, cell migration, and cell rotation. When used for cell rotation, DEP technology has long been configured in such a way that several (normally four) planar electrodes are formed, like house walls, enclosing a virtual electric-field-filled chamber or cage, inside which the cell rotates. The planar electrodes can only induce 1D cell rotation about the *Z*-axis. Furthermore, the DEP effect decays sharply in spaces that have a longer distance from the electrode edge, so the effective working area for planar electrodes is limited. In our previous study [24], we developed a new DEP configuration that enabled 3D cell rotation on a chip that is composed of four side-walls electrodes and two bottom electrodes. These six electrodes form a real chamber, inside which the cell rotates in 3D, i.e., *Z*-axis rotation is performed by applying signals to the four side-wall electrodes as what is traditionally done, *X*- or *Y*-axis rotation is performed by applying signals to the two bottom electrodes and two of the four side-wall electrodes.

The developed chip platform has the advantages of top-open and transparent structure, facilitating cell sample loading, external access to the cell sample after rotation, and non-stop observation of the cell sample during rotation. However, it still suffers from the following two major problems: (1) Like many other similar studies, we used a micropipette to place a single cell in the electrode chamber. For the bovine oocyte, which was about 120 µm in diameter, it was extremely difficult to single out a cell from the cell medium and transfer it to the chamber center. Though cumbersome, it is still acceptable to prepare cell samples using a micropipette for such large cells for a low number of experiments. In practice, however, for the majority of animal cells which are at the 10-µm scale, micropipetting of a single cell and placement is impossible. (2) The cell exhibits unstable rotation in two aspects. On the one hand, when the cell is loaded, as its initial position is not exactly in the chamber center, a non-zero DEP net force should be generated, which dynamically deviates the cell from its current position during rotation. On the other hand, it is impossible to prepare cell medium well enough that the cell would just suspend in the medium when being rotated. It was normally observed in experiments that the cell (or particle), after loading or during rotation, sink down to the bottom, which causes the whole experiment to fail because there is no way to levitate the cell again.

To overcome the above-mentioned problems, this paper reports a new design of the chip platform, which integrates one cell trap part and another 3D cell rotation part together. The cell trap part is based on hydrodynamic least flow resistance principles [25] and is similar to our previous design [26], capable of capturing one single cell from cell medium. The 3D cell rotation part is from a previous design [24], but with a simplified structure, i.e., the two bottom electrodes are integrated into one electrode. Bonded together, the two parts work separately for cell trapping and 3D rotation, respectively. With the new design, we conducted simulations to understand how to select suitable parameters for the chip, in particular, to capture a single cell, to generate a rotating electric field, and to stabilize the cell rotation process. The design and simulation results provide insightful angles to solve the existing problems and to pave the way for the next phase of our chip fabrication and experimental validation. 

## 2. Chip Design

**Figure 1:**
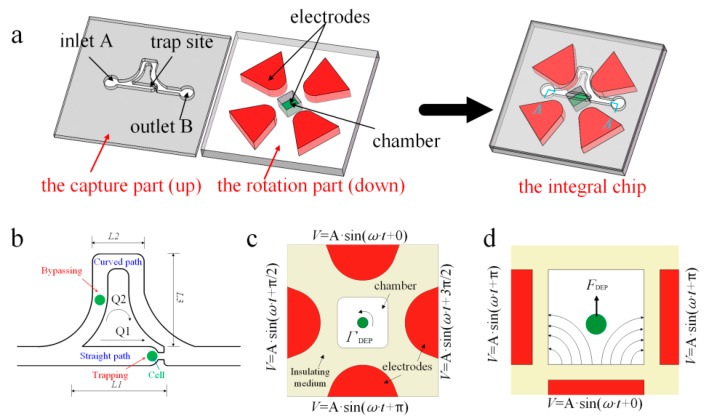
The design and theory of the integrated chip. (**a**) The separate view of the integrated model. (**b**) The schematic of the microchannel. (**c**) The top view of electrode chamber regarding dielectrophoresis (DEP) torque. (**d**) The side view of electrode chamber regarding DEP force.

The proposed chip consists of two parts: The cell capture part and the cell rotation part, bonded together, as shown in Figure 1a. The microfluidic channels on the cell capture part work to capture one cell from the cell medium, and to trap it on the trap site, then to translocate it to the chamber enclosed by the four side-wall electrodes and one bottom electrode, which are located on the cell rotation part. The side-wall electrodes can be fabricated by injecting conducting liquid or liquid metal into microchannel. Thus, the side-wall electrodes are enclosed by an insulating medium, which also forms the chamber. The microchannel of the rotation part has a special shape in order to avoid contact with the side-wall electrodes.

In our previous study [26], we proposed a passive hydrodynamic microfluidic device for high-efficiency, single-cell capture, based on the least flow resistance path principle. Here, we redesign the shape of the microchannel for integration, omitting the detailed description that was reported [25]. Simply speaking, if the flow resistance of the curved path is greater than that of the straight path, the cell medium would go through the straight path with a higher probability, such as with an electrical circuit. Therefore, when we want to trap a cell on the trap site, we simply need to satisfy the above-mentioned design condition. Figure 1b is the schematic of the microchannel. Normally, the condition is equal to that of the volume flow rate *Q*_1_ of a straight path, which is greater than the volume flow rate *Q*_2_ of the curved path before a cell gets trapped (i.e., *Q*_1_/*Q*_2_ > 1). Under this condition, the majority of the cell medium can be directed to pass through the straight path rather than curved path. When one cell is transported via medium into the trap site, it acts as a plug to dramatically increase the flow resistance of the straight path, inverting the scenario to *Q*_2_/*Q*_1_ > 1. In turn, the medium carrying other cells prefers to travel through the curved path with less flow resistance. To release the captured cell into the rotation chamber, we simply use back flow to push it away from the trap site. 

The cell rotation part originated from another study [24], but in the present model, we reduced the number of bottom electrodes, i.e., combining two bottom electrodes to one. In addition, the fabrication method of the electrodes is different from that of the previous study. The side-wall electrodes are made of conducting liquid or liquid metal, which is injected into the microchannel. In our new design, we intend to use DEP force for cell rotation stabilization and DEP torque for cell rotation. Thus, a brief introduction to these two concepts is given.

When placed in a high frequency electric field, a dielectric particle will be polarized to generate dipoles, and a Coulomb interaction will exist between the dipoles and the electric field. If the electric field is non-uniform, a net force will act on the particle to drive the particle to move toward/against the direction of the electric field maxima [27]. Similarly, a non-uniform electric field can induce torque on dipoles to make the particle rotate. 

For one case, in which two side-wall electrodes and one bottom electrode work together to generate a vertical DEP force, as shown in Figure 1c, we can use the general DEP force equation:

(1) $$F_{\text{DEP}}=2\pi R^{3}\varepsilon_{m}\text{Re}\left[K_{\text{CM}}\right]\nabla E^{2}$$

where *F*_DEP_ is the force of DEP, *R* is the radius of particle, ε_m_ is the permittivity of the medium, *E* is the electric field magnitude, Re[·] stands for the real part of a complex variable. *K*_CM_ is the Clausius-Mossotti (CM) coefficient, given by:

(2) $$K_{\text{CM}}=\frac{\varepsilon_{p}^{*}-\varepsilon_{m}^{*}}{\varepsilon_{p}^{*}+2\varepsilon_{m}^{*}}$$

(3) $$\varepsilon_{i}^{*}=\varepsilon_{i}-j\frac{\sigma_{i}}{\omega }\;\left(i=\text{m or p}\right)$$

where ε_*i*_ is the permittivity and σ_*i*_ is the conductivity of particle (*i* = p) or medium (*i* = m). The subscript of ε^*^ is p or m, which denotes the complex permittivity of the particle or medium, which are a function of the conductivity σ and the frequency ω of the electric field. *j* is imaginary unit.

In our chip, the chamber is enclosed by insulating medium, which could attenuate the electric field in the chamber; thus, a sufficiently large voltage is needed so that the electric field can penetrate the resistance of the insulating medium and induce enough DEP force and torque in the chamber.

The direction of the DEP force is related to Re[*K*_CM_] coefficient. When Re[*K*_CM_] is positive, DEP force will be a positive DEP (pDEP), which drags the particle to the high gradient electric field. In contrast, when Re[*K*_CM_] is negative, the DEP force will be a negative DEP (nDEP), which pushes the particle away from the high gradient electric field. 

If a cell suspends in a rotational electric field, it will experience a rotational torque and, thus, rotate, as shown in Figure 1d. The rotational electric field torque Γ_ROT_ is a function of the radius of the particle (*R*), the electric field strength (*E*), and the permittivity of the medium (ε_m_). Im[·] stands for the imaginary part of a complex variable.

(4) $$\Gamma_{\text{ROT}}=-4\pi R^{3}\varepsilon_{m}\text{Im}[K_{\text{CM}}]E^{2}$$

Assuming that the particle rotates at a constant angular velocity, Ω, then the hydrodynamic torque Γ_f_ arising from the Stokes drag force is given by:

(5) $$\Gamma_{f}=8\pi \eta \Omega R^{3}$$

where η is the viscosity of the medium.

In equilibrium, |Γ_ROT_| = |Γ_f_|. Combining Equations (4) and (5) we get:

(6) $$\Omega =\frac{\varepsilon_{m}}{2\eta }\text{Im}[K_{\text{CM}}]\cdot E^{2}$$

This indicates that the particle’s steady angular speed is related to the medium and the electrical properties of the particle, the electric field magnitude, and the frequency of signals; therefore, the speed and direction of the particle’s rotation can be controlled by adjusting these factors. 

## 3. Simulation and Results

### 3.1. The Placement of a Single Cell in the Rotation Chamber

To allow cell capture at the trap site and extra cell detour via the other channel, we need to simulate the design such that the volume flow rate ratio is *Q*_1_/*Q*_2_ > 1 before cell capture, and *Q*_1_/*Q*_2_ < 1 after capture. To check this condition, we build a model (*L*_1_ = 215 μm, *L*_2_ = 100 μm, *L*_3_ = 300 μm) and simulated it in COMSOL4.3b (Comsol Inc., Stockholm, Sweden) to get the flow rate distribution corresponding to the two cases, before and after cell capture. 

**Figure 2:**
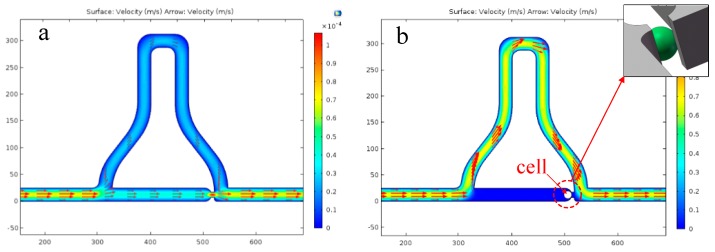
The flow rate distribution of microchannel before and after one-unit single cell be captured. (**a**) The volume rate of the straight channel is greater than that of the curved channel (*Q*_1_/*Q*_2_ > 1). (**b**) The rate distribution changed after one-unit single cell is captured (*Q*_1_/*Q*_2_ < 1). As shown in the detail figure, a cell is considered as an uncompressible solid sphere. Thus, the trap site is not completely blocked after the cell is captured, and the volume rate, *Q*_1_, decreases sharply, but is not zero.

Figure 2 clearly shows the flow rate distribution change, before and after cell capture. *Q*_1_/*Q*_2_ is equal to 2.31 before capture, so the cell will more easily pass through the straight path. After one cell is captured at the trap site, the site is blocked and the value of *Q*_1_/*Q*_2_ reduces to 0.46, then, the following cells will more easily go through the short-length curved path.

**Figure 3:**
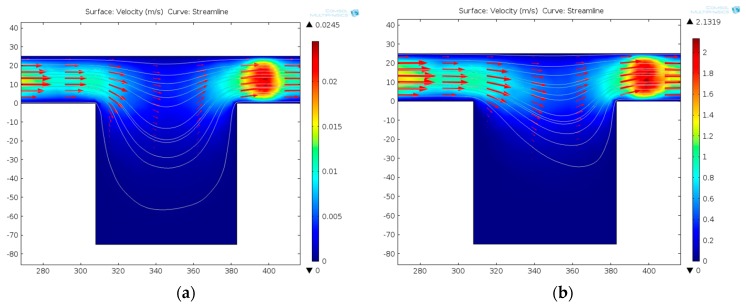

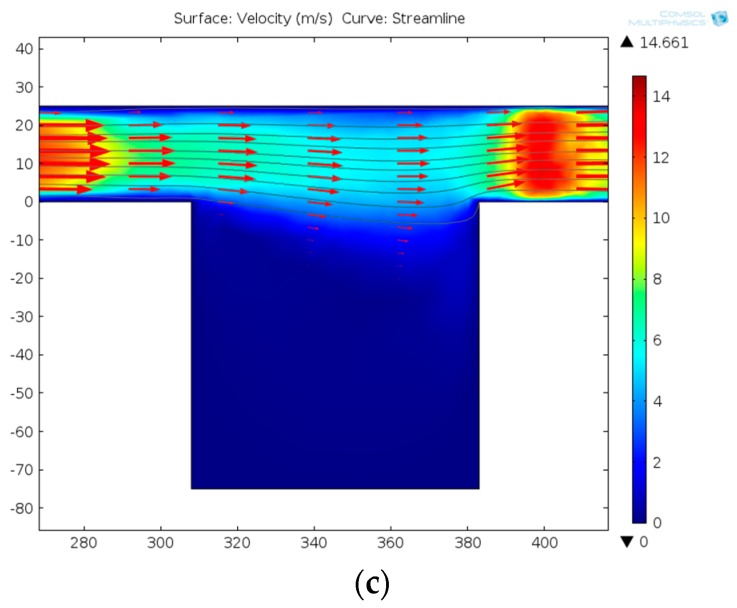
The streamline distributions of the A-A section view at different rates. As the rate increases, the streamline in the channel will be straighter, so a cell will more easily arrive at the trap site. (**a**) 5 mm/s (equivalent to the volume flow rate in microchannle 0.1875 μL/min), (**b**) 50 mm/s (equivalent to the volume flow rate in microchannle 1.875 μL/min), and (**c**) 500 mm/s (equivalent to the volume flow rate in microchannle 18.75 μL/min).

When the chip is used with the cell capture part on top, the straight path of the microchannel sits above a chamber, which will affect the fluidic streamline and direct the cell to the chamber directly before arriving at the trap site. To prevent this from happening, determining a suitable flow rate range is required. According to simple calculations, when the flow rate is less than 80 m/s, the Reynolds number is less than 2000 and the flow is still laminar. We, thus, chose three orders of magnitude for the flow rate, 5, 50 and 500 mm/s, for simulating streamline distribution in the channel. As shown in Figure 3, the faster the flow rate is, the more focused the streamlines become in the straight channel. This implies that a faster fluid flow is preferred in order for the cell to not fall into the chamber. However, the fast fluid flow can produce high shear stresses at narrow constrictions, which squeeze cells through the constrictions with high-degree deformations [26].

### 3.2. Making Cell Have Stable 3D Rotations

#### 3.2.1. Dynamic Central Position Adjustment for a Self-Adapted Cell

**Figure 4:**
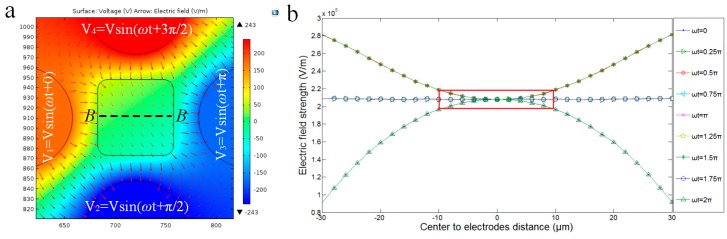
The electric field distribution in the electrode chamber. (**a**) The top view of the electric field distribution of the chamber. (**b**) The electric field strength of the center cutline, B–B, of the chamber. The red solid rectangular box indicates that the field distribution exhibits about 20% variation and the cell in the region can maintain a stable rotation.

For the four-electrode configuration to produce a rotating electric field, the electric field distribution (Figure 4a) on the *X*-*Y* plane is actually not stable or uniform. The electric field strength distribution along the center cutline, B–B, of the chamber in one AC signal period is time-varying (Figure 4b). The field strength has the smallest deviation in the central region, in particular, in the region with a size matching a 20-μm-scaled cell (indicated by the red solid rectangular box); the field distribution exhibits about 20% variation. This relatively stable region is always desirable for the cell in order to maintain a stable rotation with a steady speed and rotating point. However, to put a single cell into this restricted region is impossible, considering that the stable area only accounts for 6.25% of the whole chamber area, and significant dislocation of a cell in the central region is always the case. This dislocation effect of the cell must be compensated for by applying additional signals to the electrodes.

**Figure 5:**
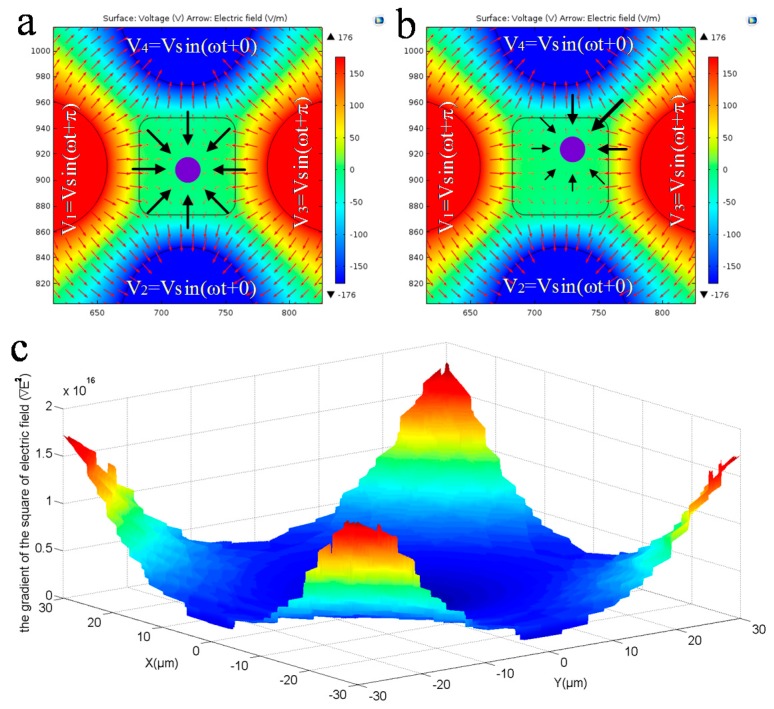
Trapping a cell in the central region of the electrode chamber. (**a**) The top view of the electric field distribution of the electrode chamber and the cell is in a balanced state. (**b**) The cell deviates from the balanced position, and the resultant force is not zero. (**c**) The distribution of the gradient of the square of the electric field in the electrode chamber.

We therefore propose to use nDEP to adaptively center the cell on the *X-Y* plane, by taking advantage of the fact that nDEP force will push the particle away from the region with a higher electric potential gradient. To do so, signals with a phase shift of π are applied to the adjacent side-wall electrodes. According to the electric field gradient simulation results (Figure 5a), the DEP forces will all point to the center with a lower gradient. If a particle or cell sits well in the center point, it would bear a net zero DEP force and, thus, be stable. 

However, if the cell deviates its position at the chamber center, the resultant net DEP force will be non-zero and will direct to the center point, which pushes the cell back to the center, where the resultant force is zero (Figure 5b). Note that the gradient distribution inside the chamber is not uniform (Figure 5c), i.e., it is rather uniform in the central region, while nonuniform obviously near the electrode edges. This indicates that if a cell gets closer to the electrodes, the net DEP force becomes greater and accelerates to push it back to the center, which is desirable in experiments. 

#### 3.2.2. DEP Force Analysis for Balancing Gravity

**Figure 6:**
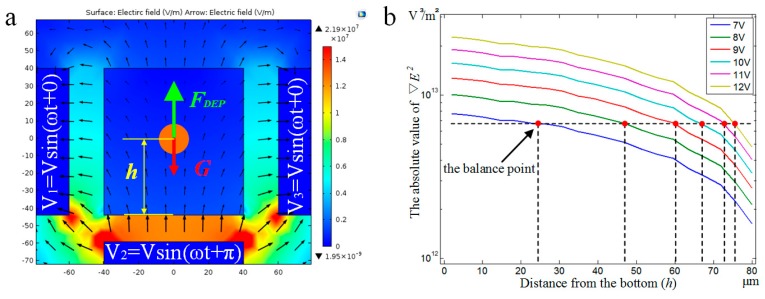
Levitation of cell to overcome the sinking problem. (**a**) Side view of electric field strength distribution of the chamber. (**b**) The value of $\left|\nabla E^{2}\right|$ for different heights of the cell and bottom electrode.

A cell must be suspended properly in the medium in order to rotate it stably. Normally, cells have a slightly higher density than their medium and would naturally fall to the bottom surface. In principle, one can fine-tune the medium by changing the conductivity or viscosity, such that cells can suspend rather than sink, but this would cause serious problems, such as DEP overheating or a high Reynolds number. Even with a proper medium, after some time of *X*- or *Y*-axis rotation the cell was observed to drop in practice. To suspend a cell somewhere in the middle of the chamber, based on the new structure with one bottom electrode, we propose to apply signals with a phase shift of π on two side-wall electrodes and the bottom electrode. The signal configuration can produce an upward electric field gradient in a vertical direction; thus, a net nDEP force is generated to counter cell gravity (Figure 6a).

Assume that the cell is loaded with the gravity, nDEP force, and buoyancy in the *Z*-axis. When the DEP force, plus buoyancy, equal gravity, the cell will be able to suspend in the buffer solution. The equation is described as:

(7) $$F_{\text{DEP}}+F_{\text{bouyancy}}=G$$

where *F*_DEP_ is the force of DEP, *F*_bouyancy_ is bouyancy force and *G* is the weight of the cell.

After some mathematical manipulation, we have $2\pi R^{3}\varepsilon_{m}\text{Re}[K_{\text{CM}}]\nabla E^{2}=\frac{4}{3}\pi R^{3}(\rho_{c}-\rho_{m})g$, which gives:

(8) $$\nabla E^{2}=\frac{2(\rho_{c}-\rho_{m})g}{3\varepsilon_{m}\text{Re}[K_{\text{CM}}]}$$

where ρ_c_ is the density of cell, ρ_m_ is the density of medium and *g* is gravitational acceleration.

Using the parameters summarized in Table 1, we can calculate a critical value for $\nabla E^{2}$, which is −6.72 × 10^12^.

Assume that the cell is centered on the *X*-*Y* plane. $\nabla E^{2}$ is dependent on its height relative to the electrode, electrode geometry, and applied signals (amplitude and frequency), and can be obtained by simulations with a combination of these parameters. For compactness, we only show here the simulation results for one fixed frequency of 1 MHz ($\text{Re}[K_{\text{CM}}]$ = −0.8 under the frequency) in Figure 6b, in which the force equilibrium points are highlighted for a range of voltage amplitudes. These curves provide a guideline for configuring the electrode signals in practice. For example, when the amplitude is 7 V, an equilibrium point (i.e., cell centroid) is located 24 μm above the bottom, leaving about a 20-μm gap between the cell and the bottom surface, which allows the cell to rotate freely in its suspended status. 

#### 3.2.3. Electric Field Analysis for Pitch Rotation of Cell

**Figure 7:**
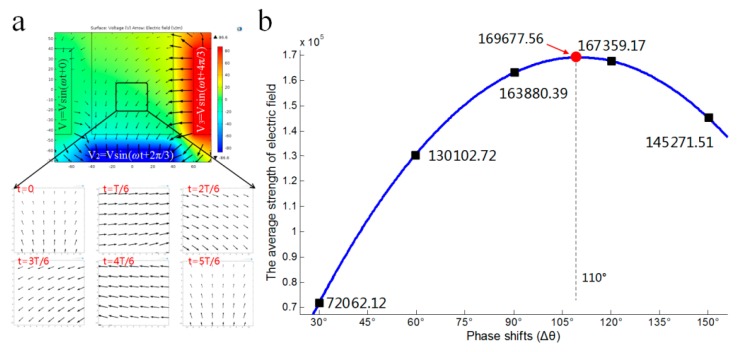
The electric field distribution of rotation about the *X*-axis. (**a**) The electric field distribution of the electrode chamber with one bottom electrode. (**b**) The average strength of the electrical field of one period within the 10-μm-square region at the center of the chamber.

The previous design with two bottom electrodes can make a cell rotate about the *X*-axis by applying signals with 5π/18 phase shifts on the two bottom electrodes. The use of the two bottom electrodes complicates device fabrication and rotation control. In our new design, we found it useful to combine the two bottom electrodes into one, where the key factor is to choose the correct phase shift between the three side-wall and bottom electrodes. Figure 7a shows the rotating electric fields generated by the newly-design chip. Though the field rotates unevenly in one cycle as well, the previous design has been validated and the time-varying rotation speed of the field gives rise to little effect on cell rotation stability. Thus, this design should have a high probability of working in the same manner. Note that the new design also provides the flexibility to change the rotating electric field direction, simply by reversing the signals applied to the three electrodes. In addition, this configuration induces rotation in the plane cross-sectioning the three powered electrodes, leaving the other two side-wall electrodes floating electrically. 

Through simulations, we also found that the electric field strength can be affected by the phase shift between electrodes. Suppose that the phase shift is Δθ, using different values in the simulations, we obtained a curve relating the field strength to the phase shift for the chamber center point (Figure 7b). It was found that the strength reached a peak at Δθ = 110°. This suggests that, even keeping the signal amplitude and frequency constant, the electric field strength can be maximized by selecting a proper phase shift. 

## 4. Fabrication of the Device

**Figure 8:**
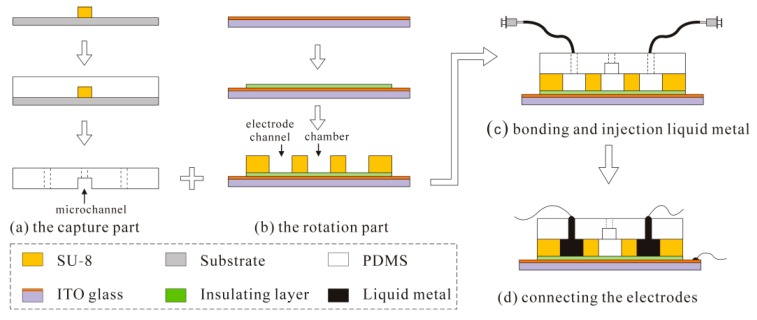
The fabrication process of the biochip. (**a**) The fabrication process of the capture part, (**b**) The fabrication process of rotation part, (**c**) Bonding and injection liquid metal, and (**d**) Connecting the electrodes.

This paper aims to design and simulate a biochip platform that works for single cell capture and 3D cell rotation. Currently, efforts have been made to fabricate the two parts of the chip. Figure 8 shows the fabrication process of the biochip. For fabricating the cell capture part, we relied on streamlined soft photolithography technology, which involves fabricating an SU-8 mold first and then replicating the mold by pouring polydimethylsiloxane (PDMS) and a cure agent into the SU-8 mold. After curing the PDMS, it is peeled off the mold to get the cell capture part. The cell rotation part was made by patterning channels on an SU-8 layer above an indium tin oxide (ITO) glass substrate. In order to avoid circuit shorts, an insulating layer (BN303-30, Kempur (Beijing) Microelectronics Inc., Beijing, China) was coated onto the ITO glass. The four V-shaped channels are placed in rotational array and the heads orthogonally enclosed a small region with their V-tips. The rotation chamber is formed inside this region. The two parts are bonded together, and two holes are punched as an inlet and outlet for the cell capture part to work. Eight holes are for the V-shaped channels to fill with liquid metal (Ga-In alloys, melting point: 60 °C), which are then solidified as the side-wall electrodes. Finally, the biochip is connected to the cell buffer solution medium and electrical connections are wired from the electrodes to the external potential supply of AC signals.

**Figure 9:**
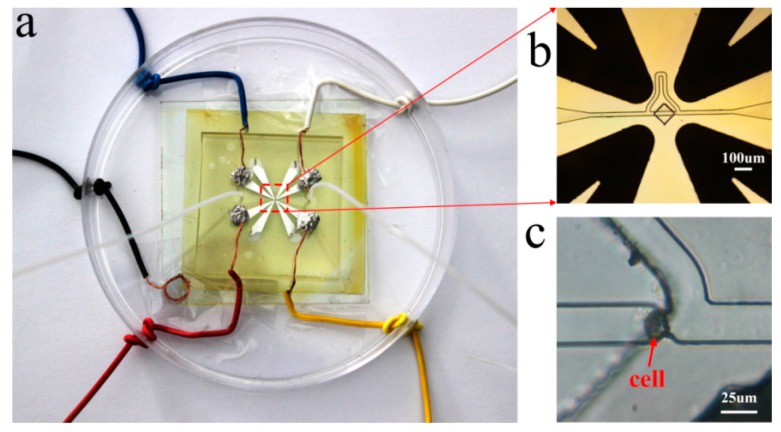
(**a**) The biochip platform. The biochip is connected to the cell buffer solution medium and electrical connections are wired from the electrodes to the external potential supply of AC signals. (**b**) Close-up view of the electrodes and channel. (**c**) Snapshot of one captured cell in the trap site.

Figure 9 shows the fabricated chip and cell capture results. The size of the microchannels and the rotation chamber can be changed to accommodate different cell sizes. The cell density in the buffer solution medium can be very low to avoid overcrowded cells from blocking the channel. Hydrostatic forces are preferred to drive the cell buffer solution medium in order to avoid high-degree deformations on the captured cell.

## 5. Experimental Demonstration and Discussion

The experimental procedure can be divided into six steps.

- Driving the solution into the capture part, and cells prefer to go through the straight line.
- One cell is captured at the trap site and the following cells flow into the curved path bypass though the straight path.
- Back and low rate flow drives the cell into the chamber.
- Apply AC signals to make the cell self-adapt to the central position and suspend.
- Apply another signals configuration to make the cell perform a 3D rotation.
- Levitate and recycle the cell. Figure 9c is a picture of one single cell captured at the trap site.

Though the above simulation results show that the cell in the rotation chamber can be stabilized using the DEP force in the space, and rotated in 3D. In practice, extra care should be taken to avoid DEP-effect interferences caused by two or more configurations of signals applied to the electrodes. This is because the real and imaginary parts of the *K*_CM_ correspond to the DEP force and torque, respectively, and any signal applied to the electrodes can induce the DEP force and torque simultaneously. In order to minimize the effect cross-talking disruption, signals of different configurations should be applied, one-by-one, rather than in parallel. How to exactly control the time sequencing of the signals need to be determined in a future experiment.

HeLa cells of about 12 μm diameter were used in experiment. Figure 9c is a picture of one single cell captured at the trap site. According to the theory of DEP, the solution medium should have low conductivity. Deionized (DI) water is a feasible and low-cost medium. The parameters of the HeLa cells and DI water are listed in Table 1, and the viscosity of DI water is 0.89 mPa·s. Using these parameters, we are able to simulate the rotation rate with respect to the AC signal frequency and voltage, and, thus, compare with experimental results. 

**Figure 10:**
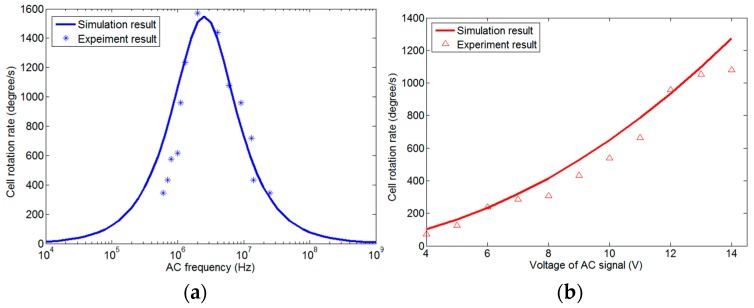
Comparison between simulated and experimental rotation rates of HeLa cells. (**a**) The relationship between the cell rotation rate and the AC frequency at *V*_p-p_ = 7 V. (**b**) The relationship between the cell rotation rate and the voltage at *f* = 600 kHz.

At present, we have obtained the experimental results for in-plane rotation. Figure 10 is the comparison of simulated and experimental rotation rates. Figure 10a shows cell rotation rate versus frequency. The rotation rate spectrum has a peak value at *f* ≈ 2 MHz, and the curve is very close to the simulation result. Figure 10b shows the cell rotation rate versus equivalent voltage in the chamber. As indicated by Equation (6), the rotation rate obtained from the experiment is nearly parabolic to the amplitude of the voltage. Thus, Figure 10 shows very good agreement between the simulation and experiment. We also observed that a cell can rotate in two directions about each axis by simply reversing the phase shifts of the signals on the neighboring electrodes. The speed of rotation can be mainly controlled by adjusting the amplitudes and the frequencies of the AC signals.

Base on the above analyses, chip fabrication, and preliminary experiments, future work should focus on experiments to accurately manipulate single cells. 

## 6. Conclusions

A rotating electrode chip combined with a microfluidic layer is presented in this paper. Numerical simulations were conducted to guide AC signal configurations and device optimization, leading to a facile trapping and lasting and steady cell manipulation. Single cells can be placed into the electrode chamber easily using the cell rotation part. By changing and adjusting the AC signal configurations, the cell can be manipulated steadily, avoiding deviation and sinking problems. In summary, this work, not only changed and improved the efficiency of the cell placing, but is also able to stably rotate the cell in 3D. This novel chip can self-adapted and trap cells efficiently, and prevent the sinking problem, which improves the efficiency and quality of the whole experiment. 

## Figures and Tables

**Table 1:** Parameter constants of material and samples.

Item	Relative Permittivity (ε)	Conductivity (σ) S/m
Insulating material	2.55	3 × 10^−^^12^
Cell	70	0.5
Buffer medium (DI water)	78	5.5 × 10^−^^3^

## References

[1] Soffe R., Tang S., Baratchi S., Nahavandi S., Nasabi M., Cooper J.M., Mitchell A., Khoshmanesh K. (2015). Controlled rotation and vibration of patterned cell clusters using dielectrophoresis. Anal. Chem..

[2] Jo Y., Shen F., Hahn Y., Park J., Park J. (2016). Magnetophoretic sorting of single Cell-Containing microdroplets. Micromachines.

[3] Gascoyne P., Shim S. (2014). Isolation of circulating tumor cells by dielectrophoresis. Cancers.

[4] Rao L., Cai B., Wang J., Meng Q., Ma C., He Z., Xu J., Huang Q., Li S., Cen Y. (2015). A microfluidic electrostatic separator based on pre-charged droplets. Sens. Actuators B Chem..

[5] Yasukawa T., Yamada J., Shiku H., Mizutani F., Matsue T. (2013). Positioning of cells flowing in a fluidic channel by negative dielectrophoresis. Sens. Actuators B Chem..

[6] Han S., Joo Y., Han K. (2013). An electrorotation technique for measuring the dielectric properties of cells with simultaneous use of negative quadrupolar dielectrophoresis and electrorotation. Analyst.

[7] Liang Y.L., Huang Y.P., Lu Y.S., Hou M.T., Yeh J.A. (2010). Cell rotation using optoelectronic tweezers. Biomicrofluidics.

[8] Cen E.G., Dalton C., Li Y., Adamia S., Pilarski L.M., Kaler K.V.I.S. (2004). A combined dielectrophoresis, traveling wave dielectrophoresis and electrorotation microchip for the manipulation and characterization of human malignant cells. J. Microbiol. Meth..

[9] Hosseini S.M., Hajian M., Moulavi F., Asgari V., Forouzanfar M., Nasr-Esfahani M.H. (2013). Cloned sheep blastocysts derived from oocytes enucleated manually using a pulled pasteur pipette. Cell. Reprogr..

[10] Hosseini S.M., Moulavi F., Asgari V., Shirazi A., Abazari-Kia A.H., Ghanaei H.R., Nasr-Esfahani M.H. (2013). Simple, fast, and efficient method of manual oocyte enucleation using a pulled Pasteur pipette. In Vitro Cell. Dev. Biol. Anim..

[11] Kirkham G.R., Britchford E., Upton T., Ware J., Gibson G.M., Devaud Y., Ehrbar M., Padgett M., Allen S., Buttery L.D. (2015). Precision assembly of complex cellular microenvironments using holographic optical tweezers. Sci. Rep..

[12] Blomqvist C., Dinér P., Grøtli M., Goksör M., Adiels C. (2014). A Single-Cell study of a highly effective Hog1 inhibitor for in situ yeast cell manipulation. Micromachines.

[13] Vergucht E., Brans T., Beunis F., Garrevoet J., De Rijcke M., Bauters S., Deruytter D., Vandegehuchte M., Van Nieuwenhove I., Janssen C. (2015). In vivo X-ray elemental imaging of single cell model organisms manipulated by laser-based optical tweezers. Sci. Rep..

[14] Chen L., Offenhäusser A., Krause H. (2015). Magnetic tweezers with high permeability electromagnets for fast actuation of magnetic beads. Rev. Sci. Instrum..

[15] Ebrahimian H., Giesguth M., Dietz K.J., Reiss G., Herth S. (2014). Magnetic tweezers for manipulation of magnetic particles in single cells. Appl. Phys. Lett..

[16] Phurimsak C., Tarn M., Pamme N. (2016). Magnetic particle Plug-Based assays for biomarker analysis. Micromachines.

[17] Adhikari A.S., Chai J., Dunn A.R. (2012). Multiplexed single-molecule force proteolysis measurements using magnetic tweezers. J. Vis. Exp..

[18] Liang W., Wang S., Dong Z., Lee G., Li W.J. (2012). Optical spectrum and electric field waveform dependent optically-induced dielectrophoretic (ODEP) micro-manipulation. Micromachines.

[19] Wang L., Flanagan L.A., Jeon N.L., Monuki E., Lee A.P. (2007). Dielectrophoresis switching with vertical sidewall electrodes for microfluidic flow cytometry. Lab Chip.

[20] Tang S., Yi P., Soffe R., Nahavandi S., Shukla R., Khoshmanesh K. (2015). Using dielectrophoresis to study the dynamic response of single budding yeast cells to Lyticase. Anal. Bioanal. Chem..

[21] Menad S., Franqueville L., Haddour N., Buret F., Frenea-Robin M. (2015). NDEP-driven cell patterning and bottom-up construction of cell aggregates using a new bioelectronic chip. Acta. Biomater..

[22] Zhang P., Ren L., Zhang X., Shan Y., Wang Y., Ji Y., Yin H., Huang W.E., Xu J., Ma B. (2015). Raman-Activated cell sorting based on dielectrophoretic Single-Cell trap and release. Anal. Chem..

[23] Graham D.M., Messerli M.A., Pethig R. (2012). Spatial manipulation of cells and organelles using single electrode dielectrophoresis. Biotechniques.

[24] Benhal P., Chase J.G., Gaynor P., Oback B., Wang W.H. (2014). AC electric field induced dipole-based on-chip 3D cell rotation. Lab Chip.

[25] Tan W.H., Takeuchi S. (2007). A trap-and-release integrated microfluidic system for dynamic microarray applications. Proc. Natl. Acad. Sci. USA.

[26] Jin D., Deng B., Li J.X., Cai W., Tu L., Chen J., Wu Q., Wang W.H. (2015). A microfluidic device enabling high-efficiency single cell trapping. Biomicrofluidics.

[27] Jones T.B., Jones T.B. (2005). Electromechanics of particles.

